# *Rhizobium leguminosarum* bv. *viciae*-Mediated Silver Nanoparticles for Controlling Bean Yellow Mosaic Virus (BYMV) Infection in Faba Bean Plants

**DOI:** 10.3390/plants12010045

**Published:** 2022-12-22

**Authors:** Ahmed Abdelkhalek, Yara Yassin, Ahmed Abdel-Megeed, Kamel A. Abd-Elsalam, Hassan Moawad, Said I. Behiry

**Affiliations:** 1 Plant Protection and Biomolecular Diagnostic Department, ALCRI, City of Scientific Research and Technological Application (SRTA-City), Alexandria 21934, Egypt; 2 Plant Protection Department, Faculty of Agriculture (Saba Basha), Alexandria University, Alexandria 21531, Egypt; 3 Plant Pathology Research Institute, Agricultural Research Centre, Giza 12619, Egypt; 4 Agriculture Microbiology Department, National Research Centre, Cairo 12622, Egypt; 5 Agricultural Botany Department, Faculty of Agriculture (Saba Basha), Alexandria University, Alexandria 21531, Egypt

**Keywords:** faba bean, bean yellow mosaic virus, silver nanoparticles, *Rhizobium*, antioxidant enzyme, oxidative stress, gene expression

## Abstract

The faba bean plant (*Vicia faba* L.) is one of the world’s most important legume crops and can be infected with various viral diseases that affect its production. One of the more significant viruses in terms of economic impact is bean yellow mosaic virus (BYMV). The current study used the molecularly identified *Rhizobium leguminosarum* bv. *viciae* strain 33504-Borg1, a nitrogen-fixing bacteria, to biosynthesize silver nanoparticles (AgNPs) to control BYMV disease in faba bean plants. Scanning electron microscopy (SEM), a particle size analyzer (PSA) with dynamic light scattering (DLS), transmission electron microscopy (TEM), energy dispersive X-ray spectroscopy (EDX), and Fourier transform infrared spectroscopy (FTIR) were used to characterize the prepared AgNPs. The DLS, SEM, and TEM analyses revealed that the AgNPs were spherical and rough, with sizes ranging from 13.7 to 40 nm. The FTIR analysis recognized various functional groups related to AgNP capping and stability. Under greenhouse conditions, spraying faba bean leaves with the AgNPs (100 µg/mL) 24 h before BYMV inoculation induced plant resistance and reduced plant disease severity and virus concentration levels. Contrarily, the AgNP treatment enhanced plant health by raising photosynthetic rates, increasing the fresh and dry weight of the faba bean plants, and increasing other measured metrics to levels comparable to healthy controls. Antioxidant enzymes (peroxidase and polyphenol oxidase) inhibited the development of BYMV in the faba bean plants treated with the AgNPs. The AgNPs decreased oxidative stress markers (H_2_O_2_ and MDA) in the faba bean plants. The plants treated with the AgNPs showed higher expression levels of *PR-1* and *HQT* than the control plants. The study findings could be used to develop a simple, low-cost, and environmentally friendly method of protecting the faba bean plant from BYMV.

## 1. Introduction

The faba bean plant is considered the fourth most important crop in the world. It is a significant source of low-cost, high-protein, and high-carbohydrate food. It is regarded as one of the principal pulse crops farmed in the Arab world. It is a versatile crop crucial to rural communities’ socioeconomic well-being [1]. It serves as the main protein source for the underprivileged in certain Asian and African nations. Due to viral infection, Egypt’s yield production has been significantly reduced. Viral diseases were a significant factor in the plant’s growth limitations and significantly reduced the quantity and quality of the crop yields [2]. About 20 viruses were responsible for most of the faba bean viral infections in Egypt [3]. Bean yellow mosaic virus (BYMV) is the most dangerous plant virus, causing massive crop yield quality and productivity losses. BYMV lowers the global faba bean yield and restricts photosynthetic activity [4]. BYMV infections in faba bean plants reduced yield production by 17–59% [5]. Up to 30% of faba bean varieties in the Arabic region are susceptible to BYMV disease [6]. Transient vein chlorosis followed by a clear green or yellow mosaic, crinkling, size reduction, and deformation, are the common symptoms of BYMV [7].

Agrochemicals used for plant viral control have harmed the environment, compromised the safety of food items, poisoned farmers [8], led to the development of pesticide resistance [9], and eliminated non-target organisms and beneficial plant microbial relationships [10,11]. In agriculture, nanoparticles have been utilized to strengthen plant tolerance to plant pathogens, promote plant development, reduce the severity of diseases, raise crop yields, and suppress pathogen infection [12]. Furthermore, systemic acquired resistance (SAR) has been noted, associated with increased enzyme antioxidant activities, including peroxidase and polyphenol oxidase [13]. In recent years, numerous plant pathogens, including bacteria, fungi, viruses, and nematodes, have been successfully combatted using AgNPs [14]. Green or biological nanoparticle production techniques have been suggested as a safer alternative to physical and chemical procedures. They are easy to fabricate, inexpensive, safe, and environmentally friendly [15]. These green strategies for AgNP synthesis use microbes such as fungi, bacteria, and plant products or extracts as reductants in the manufacturing process [16]. Additionally, it has been suggested that some rhizobial strains function as biocontrol agents, potentially lowering the impact of the pesticides used in agricultural settings [17].

The current study aimed to isolate and identify a *Rhizobium* strain from faba bean plants and use its culture filtrate to biosynthesize AgNPs. Additionally, we aimed to characterize the biosynthesized AgNPs and evaluate their efficiency in controlling BYMV infection in faba bean plants in the greenhouse, including estimating physiological and growth parameters and quantifying POX and PPO enzymes and non-enzymatic oxidative stress marker (H_2_O_2_ and MDA) kinetics. Moreover, we intended to measure the expression levels of pathogen-related protein 1 (*PR-1*) and hydroxycinnamoyl-CoA quinate hydroxycinnamoyl transferase (*HQT*) genes. Additionally, we aimed to detect the bioactive compounds in the used culture filtrate using GC-MS.

## 2. Materials and Methods

### 2.1. Virus Isolate

The BYMV isolate (BY33504-Alx1) used in this study was previously characterized and accessioned (OM863965) [2]. The virus isolate was maintained on the faba bean plant under controlled conditions. 

### 2.2. Isolation and Identification of the Rhizobial Isolate

**Figure 1:**
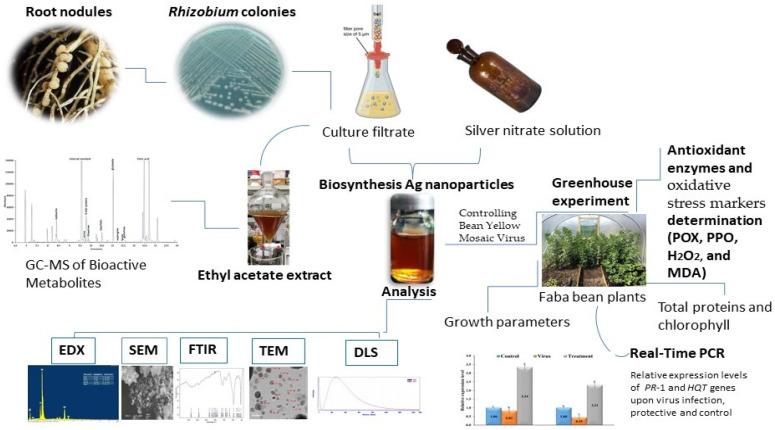
A schematic diagram summarizing the experimental study.

A schematic diagram was established to summarize the experimental study, and this is shown in Figure 1. Samples of faba bean root nodules were collected from farmers’ fields at Borg El-Arab City, Alexandria Governorate, Egypt. The nodules’ surfaces were disinfected using ethanol at a 70% concentration and 3% hydrogen peroxide [18]. Yeast extract mannitol agar (YEMA) plates were used to isolate the rhizobial bacterial strains. One isolate was selected according to pre-experiment results (data not shown). The selected isolate was inoculated in a 50-mL flask of YEM broth, incubated at 28 °C for 48 h with shaking at 100 rpm, and centrifuged, after which a pellet was obtained. Bacterial genomic DNA was extracted using an i-genomic BYF DNA extraction mini kit (Protocol B). The rhizobial isolate was subjected to molecular identification using a 16S rRNA gene-specific primer [19]. The reaction was set up for 20 µL, and the PCR was programmed for one cycle at 94 °C for 4 min, followed by 40 cycles of 45 s at 95 °C for denaturation, 45 s at 55 °C for annealing, and 90 s at 72 °C for elongation, plus 10 min for the final extension at 72 °C. The PCR fragment was purified and sequenced. The annotated sequence was deposited in GenBank and contrasted with isolates that had already been reported.

### 2.3. Characterization of Biosynthesized Silver Nanoparticles 

The rhizobial strain was inoculated into YEM broth and cultured for 48 h at 28 °C with 100 rpm shaking. The culture growth was centrifuged at 10,000 rpm for 10 min to collect the bacterial supernatant. Silver nanoparticles (AgNPs) were biosynthesized according to the method optimized by Heflish et al. [20]. After dissolving 0.0338 g of AgNO_3_ in 100 mL of distilled water, the final aqueous solution concentration was 2 mM. By adding 10 mL of bacterial supernatant to 90 mL of freshly prepared AgNO_3_ solution (2 mM), after thoroughly combining the ingredients until a noticeable color change was seen, the AgNO_3_ was ultimately reduced into biosynthesized AgNPs. The AgNPs were pelleted by centrifuging for 20 min at 10,000 rpm, then rinsed three times with sterile distilled water and washed with 70% ethanol to remove any contaminants. The produced AgNPs were dried in an oven at 50 °C. The dried AgNPs were subjected to additional physical and biological characterization using a scanning electron microscope (SEM), a particle size analyzer (PSA) with dynamic light scattering (DLS), a transmission electron microscope (TEM), energy dispersive x-ray spectroscopy (EDX), and Fourier transform infrared (FTIR). The morphology and shape of the AgNPs were examined using the SEM (JSM-6360 LA, JEOL, Tokyo, Japan) at 10 kV [20]. The particle size analyzer (Zetasizer Nano ZS, Malvern Instruments, Inc., Malvern, UK) instrument was used to analyze the particle size distribution using dynamic light scattering (DLS). The functional groups responsible for the silver nanoparticles were analyzed using an FTIR-8400S (SHIMADZU, Tokyo, Japan) in the wavelength range 4000–400 cm^−1^ [20]. All the characteristics were measured with the samples in solution form. The TEM (JEOL JEM 2010, Akishima, Tokyo, Japan) analysis was performed to obtain the morphology, size, and shape of the AgNPs and was prepared by drop-casting an aqueous bio-reduced solution on a copper TEM grid coated with carbon. Prior to the examination, the samples were dried under an infrared lamp. The TEM images were captured at a 200 kV accelerating voltage [21]. An EDX attached to a TEM was used to determine the elemental composition of the samples.

### 2.4. Greenhouse Experiment for Bionanoparticle Foliar Applications against BYMV Virus

Three virus-free seeds (faba bean, Sakha 4 cultivar) were sown in a pot of sand and clay in an equal ratio (1:1) with sterile soil. The experimental design was set up for three treatments. Each treatment involved three pots; each pot contained three plants; all treatments were administered three times. After 20 days of germination, the treatments were as follows: the first treatment included faba bean plants treated with virus inoculation buffer (control treatment). The second treatment included virus-inoculated faba bean plants only (BYMV treatment). The third treatment involved foliar spraying of the faba bean plants with AgNPs at a concentration of 100 µg/mL 24 h before BYMV inoculation (AgNP treatment/protective treatment). The mechanical inoculation of the faba bean leaves with the BYMV isolate was performed as follows: the previously infected faba bean leaves (BYMV inoculum) were pulverized in a mortar with 0.1–0.5 M phosphate buffer, pH 7.0, including 0.5% 2-mercaptoethanol at a ratio of 1:5 (W:V). Carborundum was applied to the treated leaves before mechanical inoculation. The faba bean plants were placed in greenhouse cages to protect them from flies, and daily symptom development observations were made.

#### 2.4.1. Determination of Total Chlorophyll in Plants

After 20 days following viral inoculation, the chlorophyll in the faba bean plants was determined. In brief, 5 mL of 80% acetone was added to 0.5 g of leaves, ground, and incubated at 4 °C overnight. Next, the mixture was spun at 4000 rpm for 20 min. The absorbance readings (Ar) at 645 nm and 663 nm for chlorophyll (chlr) a and b were measured with a Beckman spectrophotometer (DU730, Beckman Coulter Inc., Brea, CA, USA) using a blank solution of 80% acetone. According to Strain and Svec [22], concentrations of chlr a, b, and total chlr were calculated using the following equations:

Chlr a (mg/mL) = (11.64 × Ar 663) − (2.16 × Ar 645)

Chlr b (mg/mL) = (20.97 × Ar 645) − (3.94 × Ar 663)

Total chlr (mg/mL) = a + b

#### 2.4.2. Determination of Total Proteins

To determine the total proteins, the dried leaf powder (0.1 g) was combined with 5 mL of borate buffer (28.63 g of B(OH)_3_, 29.8 g of KCl, and 3.5 g of NaOH/L, pH 8.0). The mixture was kept in the fridge overnight and then spun for 10 min at 4000 rpm. Next, 3 mL of Coomassie brilliant blue (CBB) reagent (100 mg of CBB was dissolved in 50 mL of 95% ethyl alcohol and 100 mL of 85% H_3_PO_4_, and distilled water was then added until it reached 1 L) was combined with 0.1 mL of supernatant and mixed well for 2 min before being measured at an absorbance of 595 nm. A calibration curve was made using bovine serum albumin as the reference protein, and the concentration of total soluble proteins was calculated as mg/g of dry matter (DM) [23].

### 2.5. Estimation of Malondialdehyde (MDA) and Hydrogen Peroxide (H_2_O_2_)

#### 2.5.1. Malondialdehyde (MDA)

Malondialdehyde (MDA), a product of the peroxidation of unsaturated fatty acids, was quantified using the method of Heath and Packer [24]. First, 0.5 g of the sample’s fresh leaves were pulverized in a mortar with 10 mL of 5% trichloroacetic acid (TCA). The homogenate was spun at 4000 rpm for 15 min, and the supernatant was collected. An equal volume of the supernatant and 0.67% thiobarbituric acid (TBA) were combined. The combination was heated at 100 °C for 15 min and then immediately ice cooled. At 532 nm and 600 nm, the absorbance was measured. With an extinction coefficient of 155 mM^−1^ cm^−1^, the amount of lipid peroxidation is represented as mM/g FW (fresh weight).

#### 2.5.2. Hydrogen Peroxide (H_2_O_2_) 

To estimate the presence of H_2_O_2_, the Velikova method [25] was used. First, 0.5 g of fresh leaves and 5 mL of 0.1% (*w*/*v*) TCA were ground in a mortar. The mixture was then spun at 12,000 rpm for 15 min. Afterward, 500 µL of the supernatant was mixed with 1 mL of 100 mM KI and 500 µL of 10 mM phosphate buffer. After mixing, the absorbance was measured at 390 nm. Using a value of 0.28 mM^−1^ cm^−1^, the amount of H_2_O_2_ was estimated as µmoL/g FW.

### 2.6. Estimation of Antioxidant Enzymes

Fresh plant material weighing 0.5 g was combined and mixed with 5 mL of a 0.05 M sodium phosphate buffer (pH 7.0). The mixture was then centrifuged at 3000 rpm for 15 min, and the resulting supernatant was used for enzyme assays [26].

#### 2.6.1. Peroxidase (POX)

Peroxidase activity was determined according to the Kato and Shimizu method [27]. At 480 nm, the enzyme was calculated using the formula E = 26,600 µM^−1^ cm^1^ and expressed as µM/g FW. The reaction mixture included 0.1 mL of the supernatant extract, 1.4 mL of guaiacol (7.2 mM), 1.4 mL of H_2_O_2_ (11.8 mM), and 3 mL of sodium phosphate buffer (pH 5.8).

#### 2.6.2. Polyphenol Oxidase (PPO)

The activity of PPO was assessed using the method described by Kumar and Khan [28]. The mixture contained 500 µL of extract, 1 mL of pyrogallol (0.02 M), and 2 mL of phosphate buffer (pH 6.0). After 5 min, 1 mL of H_2_SO_4_ (2.5 M) was added to the homogenate to stop the reaction. The enzyme was measured using the formula 26.40 M^−1^ cm^−1^ at 420 nm and expressed as µM/g FW.

### 2.7. Total RNA Extraction and cDNA Synthesis 

The RNeasy Plant Mini Kit was used according to manufacturer instructions to extract the total RNA (QIAGEN, Hilden, Germany). The RNA was measured using a UV-Vis spectrophotometer (DU730, Beckman Coulter Inc., Brea, CA, USA). The RNA (2 µg) was converted into cDNA with the M-MuLV RT enzyme, Cat. No. K1691 (Thermo Fisher Scientific, Waltham, MA, USA), as described previously [29]. The reactions were carried out in a QIAGEN qPCR (Rotor-Gene Q, Hilden, Germany), in accordance with the method used by Abdelkhalek et al. [30]. The cDNA was reserved as a qRT-PCR template.

### 2.8. Quantitative Real-Time PCR

A reaction volume of 20 µL containing 10 µL SYBR mix, 1 µL of cDNA template, 1 µL of each primer, and 7 µL of molecular grade water, was used in the qRT-PCR. The transcription levels of the target genes (*PR-1*, *HQT,* and BYMV-CP) were assessed. The 18S rRNA and *β-actin* genes were employed as internal reference genes (Table 1). The qRT-PCR reaction program was set for 40 cycles of 95 °C for 15 s, 60 °C for 30 s, and 72 °C for 30 s, linked after three minutes at 95 °C [31]. The relative transcriptional levels were measured using the 2^−ΔΔCt^ algorism method [32]. The fold changes in gene transcription levels were computed based on the reference gene. All experiments were performed three times. 

### 2.9. GC-MS Analysis

After 48 h of rhizobial bacteria incubation in the YEM broth media, the bacterial supernatant was collected and combined in a 1:1 (*v*/*v*) ratio with ethyl acetate as a solvent, in accordance with the method used by Abdelkhalek et al. [33]. After 20 min of vigorous shaking, the mixture was separated using a separate funnel. The ethyl acetate phase was then concentrated by evaporating at 40 °C with a rotary evaporator. Secondary metabolites and chemical substances were found in the residue, which was examined using a gas chromatography-dual stage quadrupole mass spectrometer (Focus GC-DSQ MS, Thermo Scientific, Austin, TX, USA) with a direct capillary column TG–5MS (30 m × 0.25 mm × 0.25 μm film thickness) apparatus [34]. Helium gas flowed through a carrier at a rate of 1 mL/min. The column oven temperature was initially held at 45 °C and then increased by 5 °C min^−1^ to 200 °C held for 5 min, and then increased to 300 °C with 30 increments of 5 °C min^−1^. The injector and MS transfer line temperatures were kept at 270 °C and 250 °C, respectively. The solvent delay was 3 min, and diluted samples of 1 μL were injected automatically using the autosampler AS1310 coupled with the GC in split mode. EI mass spectra were collected at 70 eV ionization voltages over the range of 40–500 *m*/*z* in full scan mode, and the ion source was set at 200 °C. The components were identified by comparison of their retention times and mass spectra with those of the WILEY 09 and NIST 11 mass spectral databases.

### 2.10. Data Analysis

The relative transcription values for each treatment were examined using the Costat software. A Tukey post-hoc test (HSD) was used to compute the differences between the means at *p* ≤ 0.05. Relative transcription values greater than 1 represent an increase in gene transcription levels (up-regulation), and those less than 1 represent a drop in transcriptional levels (down-regulation).

## 3. Results

### 3.1. Isolation and Characterization of Rhizobium Isolate

**Figure 2:**
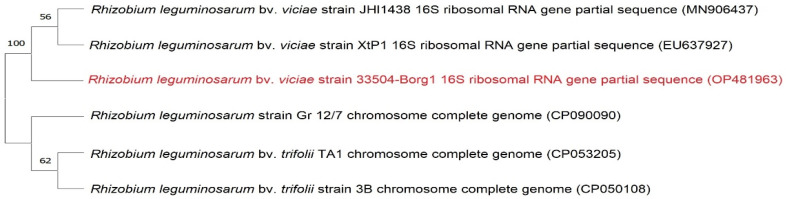
The 16S rRNA phylogenetic tree based on the sequences of *Rhizobia* isolates retrieved from the GenBank website and aligned with the Egyptian isolate *R. leguminosarum* bv. *viciae* strain 33504-Borg1 (OP481963).

From the faba bean root nodules, 42 rhizobia isolates were identified. A small amount of Congo red dye was absorbed by all the isolates, resulting in circular colonies ranging from pale pink to white with a raised smooth edge and a musky odor. The most effective rhizobia isolate from the faba bean plants was chosen and molecularly identified as *R. leguminosarum* bv. *viciae* by sequencing the amplified 16S rRNA gene. The verified sequence was submitted to GenBank, and the accession number is OP481963. In alignment with the GenBank-related sequences, the phylogenetic tree demonstrated that the 33504-Borg1 strain was 100% identical to *R. leguminosarum* bv. *viciae* strains (Figure 2). 

### 3.2. Visual Inspection, SEM, and DLS Analysis

**Figure 3:**
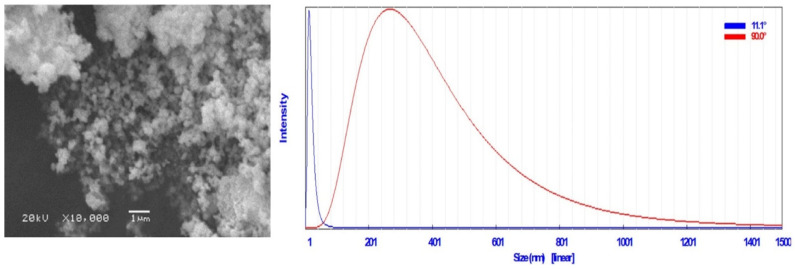
SEM image of bio-AgNPs (**left**) and their size distribution using DLS (**right**).

In this study, the color, form, and size of the AgNPs biosynthesized by the supernatant of *R*. *leguminosarum* bv. *viciae* were validated by visual inspection (observing the color changing to yellowish brown after 96 h of incubation (Figure S1)) and scanning electron microscope (SEM) examination. The AgNPs were spherical in form, with rough surfaces (Figure 3). The dynamic light scattering (DLS) analysis showed that the biosynthesized AgNPs in the aqueous medium had a particle size distribution of 13.7 nm (Figure 3). 

### 3.3. TEM and EDX Analysis

**Figure 4:**
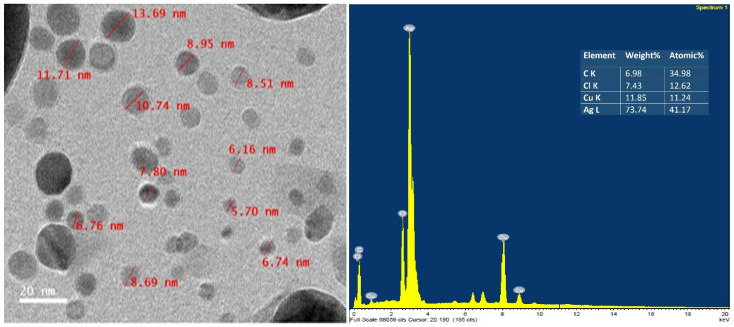
TEM image of biosynthesized AgNPs (**left**) and their EDX spectra analysis with different X-ray emission peaks (**right**).

Transmission electron microscopy (TEM) was used to characterize the nanoparticles’ size, distribution, and shape in great detail. The TEM analysis showed that the AgNPs were mono-dispersed and sphere-shaped, and that the size of the AgNPs varied from 5.70 nm to 13.69 nm (Figure 4). In addition, EDX spectra analysis results validated the presence of elemental Ag, C, Cl, and Cu in the analytical samples. A strong absorption peak appeared at about 3 KeV, confirming the metallic silver nanoparticles’ exitance (Figure 4). 

### 3.4. FTIR Analysis

**Figure 5:**
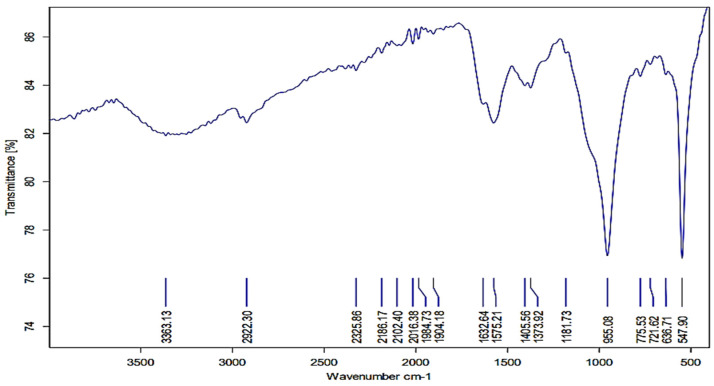
FTIR spectrum functional groups of biosynthesized AgNPs.

According to the FTIR investigation results, many of the functional groups had fluctuating absorption peaks at 547.90 cm^−1^, 955.08 cm^−1^, 1405.56 cm^−1^, 1575.21 cm^−1^, 1632.64 cm^−1^, 2922.30 cm^−1^, and 3363.13 cm^−1^ (Figure 5). The FTIR spectrogram band at 3363.13 cm^−1^ belongs to N-H. The band at 2922.30 cm^−1^ is a result of the C-H stretching of the aliphatic or methylene group. In contrast, the band peak at 1575.21 cm^−1^ is the amino peak, and the band peak at 1632 cm^−1^ indicates conjugated ketone or quinone. About 955 cm^−1^ is required for the vinyl C-H out-of-plane bend. The C-I stretch in the strong band seen at 547 cm-^1^ was caused by aliphatic iodo compounds. The band peak at 1405.56 could be a result of the stretching of the C-O group.

### 3.5. Effect of AgNPs on Growth Parameters, Total Chlorophyll Content, and Total Protein

The faba bean leaves infected by BYMV showed systemic symptoms, including venial yellowing followed by an evident yellow mosaic, crinkling, and deformity, unlike the healthy leaves. The AgNP-treated plants almost eliminated the effects of the virus on the faba bean leaves within 24 h of inoculation with BYMV. The total chlorophyll concentrations increased in the plants treated with the AgNPs (T3: 45.76 ± 0.23 mg/mL). However, the plants treated with BYMV alone showed a significant decrease in total chlorophyll accumulation levels (T2: 23.42 ± 0.21 mg/mL) (Table 2). The fresh and dry weights of the shoots and roots of the faba bean plants treated with the AgNPs were significantly higher than those of the BYMV-treated plants (Table 2). The plants treated with the AgNPs 24 h before BYMV inoculation demonstrated lower accumulated total soluble protein content (T3: 10.37 ± 0.15 mg/mL) than the plants that underwent the other treatments (T1: 13.24 ± 0.31 mg/mL and T2: 13.84 ± 0.27 mg/mL) (Table 2).

### 3.6. Effect of AgNPs on Antioxidant Enzymatic Activities (POX and PPO)

The plants treated with BYMV showed the highest peroxidase enzyme activity (T2: 1.78 ± 0.15 µmol/g FW), followed by those treated with the AgNPs (T3: 1.12 ± 0.18 µmol/g FW) and the control plants (T1: 0.88 ± 0.18 µmol/g FW). The PPO enzyme activity was increased in the plants treated with BYMV (T2: 0.27 ± 0.01 µmol/g FW) compared with the plants that underwent the other treatments (T3: 0.15 ± 0.01 µmol/g FW and T1: 0.13 ± 0.01 µmol/g FW) (Table 3).

### 3.7. Effect of AgNPs on Oxidative Stress Markers Assay (H_2_O_2_ and MDA)

MDA content was used to measure the amount of lipid peroxidation in the faba bean plants. H_2_O_2_ levels in the faba bean plants treated with the AgNPs were reduced (T3: 4.52 ± 0.07 µmol/g FW) compared with those in the plants treated with BYMV (T2: 7.56 ± 0.04 µmol/g FW) and those in the control plants (T1: 6.39 ± 0.10 µmol/g FW). MDA levels in the faba bean plants treated with the AgNPs were reduced (T3: 147.14 ± 0.10 µmol/g FW) compared with those in the plants treated with BYMV (T2: 383.98 ± 0.16 µmol/g FW) and those in the untreated faba bean plants (T1: 235.6 ± 0.10 µmol/g FW) (Table 3).

### 3.8. Effect of AgNPs on BYMV Accumulation Level and Transcriptional Levels of Defense-Related Genes

**Figure 6:**
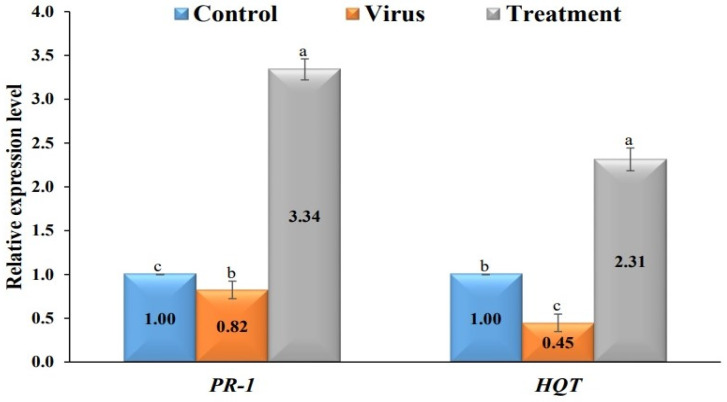
Relative expression levels of *PR-1* and *HQT* genes at 20 dpi in response to treatments. Control, healthy control plants; Virus, plants inoculated with BYMV only; Treatment, faba bean plants foliar sprayed with AgNPs 24 h before BYMV inoculation. Columns reflect the mean value from three biological replicates, and the standard deviation (SD) values are shown in the bars. Columns with the same letter above are not statistically different.

At 20 dpi, a significant increase in the relative transcriptional levels of genes (*PR-1*, *HQT*) and a decrease in the accumulation levels of BYMV were observed in the plants treated with AgNPs compared with the control plants (T1) and the plants treated with the virus (T2). The qRT-PCR results demonstrated that the virus treatment (T2) produces the highest amounts of BYMV-CP transcripts, with a relative expression of 23.68, indicating that the plant is infected with a virus. In contrast, the relative expression levels of BYMV-CP were 5.21-fold higher in the AgNP-treated plants. The low levels of BYMV found in the plants treated with the AgNPs represent an 80% reduction in viral accumulation. These findings imply that AgNPs may confer plant resistance to BYMV replication in faba bean tissues. In the current investigation, the relative transcriptional levels of the *PR-1* and *HQT* genes decreased in the faba bean plants infected with BYMV (T2), with levels 0.82-fold and 0.45-fold lower, respectively, than those observed in the control plants (T1). In contrast, the faba bean plants treated with the AgNPs 24 h before BYMV inoculation (T3) showed a significant increase in *PR-1* with relative transcriptional levels 3.34-fold, higher than those of the control plants (T1). The *HQT* gene’s relative expression level decreased in T2, with a 0.45-fold lower level than that produced by the T1 treatment. In addition, applying AgNPs (T3) increased the number of *HQT* transcripts in the faba bean plants, producing a *HQT* transcriptional level 2.31-fold higher than that observed in the control plants (T1) (Figure 6).

### 3.9. GC-MS Analysis of Bioactive Metabolites

**Figure 7:**
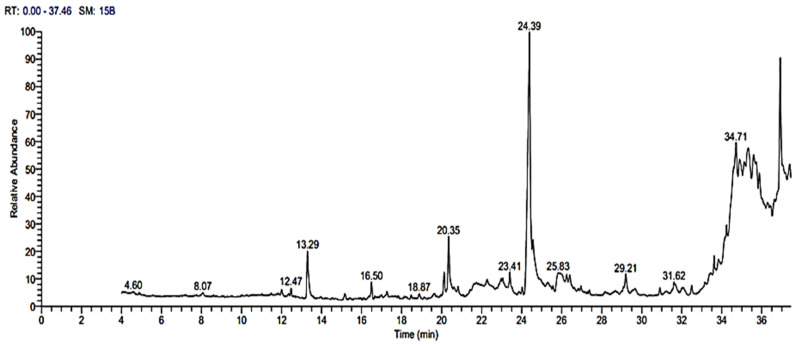
GC-MS and mass spectra of rhizobial isolate ethyl acetate extract.

A GC-MS analysis was performed to identify the bioactive components of the *Rhizobium leguminosarum* (33504-Borg 1) bacterial filtrate. The active constituents of the bacterial isolate ethyl acetate filtrate fractionated at retention times (RT) are shown in Figure 7 and Table 4. The analyzed data revealed the presence of a high relative abundance of oleic acid, with the highest peak area percentage of 20.23% at RT 24.39 min, followed by 4H-1-benzopyran-4 one, 2-(3,4-dimethoxy phenyl)-3, 5-dihydroxy-7-methoxy- (5.66%) at RT 34.72 min, and 1-hexadecanol, 2-methyl- (4.48%) at RT 25.82 min. According to mass spectrometry libraries, other constituents differed in retention time and peak area detected and quantified.

## 4. Discussion

Plant diseases, particularly viral plant infections, cause significant crop losses in crop production across the world, creating serious issues for food security [35]. The application of AgNPs synthesized by *Rhizobia* as biocontrol microbes for controlling viral plant infections has been limited until now. Nitrogen-fixing rhizobia have been identified as a practical tool for improving plant growth and crop production [36,37]. In agriculture, AgNPs have been used to enhance plant resistance to plant microbes, but the methods used for AgNP synthesis are unsafe. The current study uses a biosynthesis method which includes the supernatant of *Rhizobium leguminosarum* as a safe alternative to harmful physical and chemical methods. SEM, PSA, TEM, EDX, and FTIR analyses were used to characterize the AgNPs. Additionally, this study evaluated the antiviral activity of the AgNPs against BYMV on faba bean plants at 20 dpi and determined the total chlorophyll and protein contents, peroxidase (POX) activity, polyphenol oxidase (PPO) activity, H_2_O_2_ content, and MDA level in the plants. Finally, the effects of the AgNPs on BYMV accumulation and transcription of two defensive scheme genes (*PR-1* and *HQT*) at 20 dpi were studied.

It was observed that the biosynthesis of the AgNP process depends on the reduction time. In the current study, the AgNPs had an absorption peak at 540 nm, and the reaction time extended to 96 h rather than 72 h, as indicated in a study conducted by Kirubha and Alagumuthu [38]. There was no increase in the absorbance intensity after 96 h in the AgNPs, as in the results reported by Kumar and Mamidyala [39]. A typical method for determining the size distribution profile of particles in a colloidal solution is DLS [40]. DLS analysis results showed that the particle size of the AgNPs was in the same range as that of AgNPs synthesized (9–11 nm) from the supernatant of *Bacillus* sp. [41]. Thus, the bio-reduced aqueous solution could also contain silver nanoparticles generated biologically in a perfect dispersion [42]. SEM is a popular technique for assessing the surface morphology, size, and distribution of synthesized AgNPs [43,44]. SEM micrographs can also reveal the purity and polydispersity of synthesized AgNPs [45]. In our study, the SEM analysis showed that the particles were shaped like spheres with rough surfaces, which agrees with the results reported by Raj et al. [46]. Additionally, the TEM analysis showed that the AgNPs were monodispersed and sphere-shaped, as in the results reported by Nagati et al. [47], who synthesized AgNPs from *Cajanus cajan* leaf extract and proved their antibacterial activity. The AgNPs were not agglomerated, as the EDX analysis yielded highly conclusive results, confirming the silver’s existence. Due to the presence of elemental silver, it is possible to state that the obtained silver nanoparticles were of high purity, as the optical absorption peak of silver nanocrystals occurs at approximately 3 KeV. However, the other observed element ion peaks, such as C, Cl, and Cu, may have come from the grid on which the sample was prepared or residual elements from the broth medium. In the FTIR analysis, the band peak at 3363.13 cm^−1^ is referred to as N-H. Absorption peaks at 3398–3359 cm^−1^ (bonds caused by N-H stretching and amides) were similar to those shown in the results of Jagtap and Bapat [48] and Heflish et al. [20]. The absorption band at 2922.30 cm^−1^ belonged to the C-H stretching of the aliphatic or methylene group [49]. In comparison, the band peak at 1632 cm^−1^ indicates conjugated ketone or quinone [40]. The amino peak is located at 1575 cm^−1^ [20]. In addition, the strong bands seen at 547 cm^−1^ belonging to the C-I stretch were caused by aliphatic iodo compounds [50]. The absorption of a strong band at 955 cm^−1^ belonged to the vinyl C-H out-of-plane bend, according to the study of Nandiyanto et al. [51]. The weak band at 1405 cm^−1^ may be referred to as the stretching of the C-O group [52]. According to the FTIR results, several of the biological components from the *Rhizobium* extract formed a potent capping agent on the nanoparticles to help stabilize them [53].

In the greenhouse trial, the plants sprayed with the AgNPs had less severe disease symptoms and lower viral accumulation levels than the infected faba bean plants that were not treated. This result might be due to the AgNPs’ capacity to trigger plant resistance to BYMV infection, or it could be because the AgNPs affect RNA copying during viral multiplication. It is clear that the AgNPs significantly inhibit viral nucleic acid replication, as Yang et al. [54] and Cai et al. [55] have reported. Compared with the BYMV treatment, the AgNP treatment significantly enhanced the total chlorophyll and plant growth parameters, such as the weights of the faba bean shoots and roots. Similar results were obtained by Sameh [56] and Rostamizadeh et al. [57]. The significant increase in plant growth observed in our study could be attributed to the AgNPs’ ability to stimulate the synthesis of different phytohormones (gibberellins) in faba bean plants [58]. Therefore, AgNPs may activate the induced systemic resistance system in faba bean plants against BYMV infection. Our study reported the highest protein content in the faba bean plants infected with BYMV. These findings are consistent with the results of Sameh [56], which indicate that total protein increases when plants are under stress. Our findings noted a significant increase in POX and PPO activity in the T2 plants (BYMV), followed by the T3 plants. The same results were obtained by El Gamal et al. [59], who noted that POX and PPO activity increased with AgNP application. POX and PPO have been observed to inhibit the development of plant diseases and support defense systems. They are responsible for reducing the negative effects of stresses such as viruses via reactive oxygen scavenging [60].

In the present study, the faba bean plants treated with BYMV only significantly increased their H_2_O_2_ and MDA content compared with the control plants. Increasing H_2_O_2_ and MDA indicate that the plants are under significant oxidative stress, so treatment with AgNPs reduced H_2_O_2_ and MDA levels. These data agree with Mondal et al. [61] and Anthony et al. [62], who reported that viral infection increased H_2_O_2_ and MDA levels. In our study, the faba bean plants infected with BYMV (T2) had lower relative transcriptional levels of *PR-1* and *HQT* than the control plants. Interestingly, the faba bean plants treated with the AgNPs had higher transcriptional levels of *PR-1* and *HQT* with a higher relative expression level than control plants. The AgNP treatment decreased viral accumulation levels in the faba bean plants by about 80%. Salicylic acid (SA) has been a popular plant hormone-signaling molecule for regulating plant immunity activation for over two decades [63]. Induction of *PR-1*, an SA marker gene, after pathogen infection is frequently associated with SA activation [64,65]. According to various reports, SAR activation is caused by a group of pathogen-related (PR) proteins that are also effective at preventing pathogen development, multiplication, and/or spread [66]. As a result, we assume that the AgNPs contain elicitor metabolite compounds that can activate SAR and stimulate plant resistance to BYMV infection.

Secondary metabolites such as polyphenolic compounds are important for plant growth, development, and tolerance to biotic and abiotic stresses, such as viruses [67]. Chlorogenic acid (CGA), one of the most useful phenolic compounds, enhances plant resistance and suppresses pathogens such as viruses [68]. BYMV has been proven to limit CGA production within infected faba bean tissues. A key enzyme in the production of CGA is *HQT*, which catalyzes the conversion of caffeoyl-CoA into quinic acid [69]. It has been discovered that increased CGA content within plant tissues is linked to *HQT* over transcription, and vice versa [70]. Our results showed that foliar AgNP treatment 24 h before BYMV inoculation enhanced the transcription of *HQT*, resulting in the accumulation of phenolic molecules and the triggering of SAR for BYMV infection.

The GC-MS spectrum analysis revealed that the supernatant of *Rhizobium leguminosarum* 33504-Borg 1 contains many fatty acid methyl esters. Oleic acid is the major compound in *Rhizobium* ethyl acetate extract, which is one of the fatty acids that can protect plants against pathogenic microorganisms. Several studies have reported the antibacterial properties of many fatty acids derived from multiple biological sources, including algae and plants [71]. Short- and long-chain fatty acids derived from the green algae *H. pluvialis* and *S. obliques* have been shown to have antimicrobial properties against *S. aureus* and *E. coli* [72]. Another study performed by El-Gendi et al. [73] reported the presence of eicosane (long-chain) and pentadecanoic acid (unsaturated) in a *Bacillus subtilis* culture filtrate GC-MS. Eicosane, an active fatty acid found in many microorganisms, has shown significant antifungal activity against tobacco leaf spot disease [74,75], and it has also been observed in plant extract analysis [76]. Many fatty acids, such as oleic acid, modulate various responses to abiotic and biotic stresses in plants [77]. According to Cohen et al. [78], fatty acids such as oleic, linolenic, arachidonic, docosahexaenoic, eicosapentaenoic, and linoleic acids can activate the systemic potato resistance against the *Phytophthora infestans* pathogen. In the same way, TMV accumulation levels in oleic acid-treated tobacco leaves have been decreased by increasing oleic acid concentration, as reported by Lei Zhao et al. [79]. This finding suggests that oleic acid may inhibit BYMV replication in plants and confirms previous reports of fatty acid methyl ester compound activity in preventing viral replication, particularly oleic acid.

Overall, this study offers a fresh understanding of how biological agents from isolated Rhizobia synthesize AgNPs by reductive processes. The rhizobial isolates created extracellular catalysts for the synthesis of AgNPs. These extracellular substances were most likely to reduce silver ions, resulting in nanoparticle production. The nanoparticles, particularly those produced by the rhizobial isolates, demonstrated excellent in vitro antimicrobial activity against BYMV infection as well as significant antiviral activity, which is of substantial importance for many African countries as BYMV infections have the potential to reduce the yield of faba bean crops significantly. This knowledge is crucial for the future registration and labeling of AgNPs as antiviral crop protection agents, as well as for the clarification of the mechanisms underlying virus inactivation.

## 5. Conclusions

The current study has demonstrated that AgNPs biosynthesized by *Rhizobium leguminosarum* filtrate have the potential to control the bean yellow mosaic virus (BYMV). Dynamic light scattering (DLS), scanning electron microscope (SEM), and transmission electron microscope (TEM) analyses indicated that the AgNPs were spherical and rough, with sizes ranging from 13.7 nm to 40 nm. The greenhouse trial results revealed that spraying faba bean leaves with AgNPs 24 h before BYMV inoculation induced plant resistance and reduced plant disease severity and virus accumulation. In addition, it enhanced plant health by increasing the growth parameters, increasing peroxidase (POX) and polyphenol oxidase (PPO) enzymes, and decreasing oxidative stress markers (H_2_O_2_ and MDA). The AgNP treatment resulted in higher transcriptional levels of *PR-1* and *HQT* genes than in the control plants. The study findings could be a reliable source for developing easy-to-use, affordable, and environmentally friendly methods for managing and safeguarding the faba bean plant against BYMV. Nevertheless, more field research is required to verify these results and understand more fully the nanoparticle application dynamics.

## Figures and Tables

**Table 1:** Primer sequences used in thia study.

Gene	Abbreviation	Sequence
16S ribosomal RNA	16S rRNA	For: AGAGTTTGATCCTGGCTCAG Rev: AAGGAGGTGATCCAGCC
Pathogenesis-related protein-1	*PR-1*	For: TTCTTCCCTCGAAAGCTCAA Rev: CGCTACCCCAGGCTAAGTTT
Hydroxycinnamoyl-CoA quinate hydroxycinnamoyltransferase	*HQT*	For: CCCAATGGCTGGAAGATTAGCTA Rev:CATGAATCACTTTCAGCCTCAACAA
Bean yellow mosaic virus coat protein	BYMV-CP	For:GGTTTGGCYAGRTATGCTTTTG Rev: GAGAATTTAAAGACGGATA
18s ribosomal RNA.	18S rRNA	For: CATCAGCTCGCGTTGACTAC Rev: GATCCTTCCGCAGGTTCAC
Beta-actin	*β-actin*	For: TGGCATACAAAGACAGGACAGCCT Rev: ACTCAATCCCAAGGCCAACAGAGA

**Table 2:** Changes in fresh weight, dry weight, chlorophyll, and total protein in response to BYMV and AgNPs treatments.

Treatments *	Fresh Weight	Dry Weight	Chlorophyll	Total Protein
T1	41.07 a ± 3.50	7.07 ab ± 0.78	32.79 b ± 0.18	13.24 a ± 0.31
T2	31.77 b ± 1.99	5.26 b ± 0.98	23.42 c ± 0.21	13.84 a ± 0.27
T3	40.70 a ± 1.31	8.57 a ± 0.58	45.76 a ± 0.23	10.37 b ± 0.15

* T1: healthy control without virus inoculation or AgNP application; T2: plants inoculated with BYMV only; T3: plants foliar sprayed with AgNPs 24 h before BYMV inoculation. The different letters beside the numbers in each column mean that the data were significantly different at *p*-value ≤ 0.05.

**Table 3:** Responses of antioxidant enzymes and oxidative stress markers to different treatments.

Treatments *	Antioxidant Enzymes		Oxidative Stress Markers	
	POX	PPO	H_2_O_2_	MDA
T1	0.88 ± 0.18 b	0.13 ± 0.01 b	6.39 ± 0.10 b	235.60 ± 0.10 b
T2	1.78 ± 0.15 a	0.27 ± 0.01 a	7.56 ± 0.04 a	383.98 ± 0.16 a
T3	1.12 ± 0.18 b	0.15 ± 0.01 b	4.52 ± 0.07 c	147.14 ± 0.10 b

* T1 represents the healthy control without virus inoculation or AgNP application; T2 represents plants inoculated with BYMV only; T3 represents plants foliar sprayed with AgNPs 24 h before BYMV inoculation. The different letters beside the numbers in each column mean that the data were significantly different at *p*-value ≤ 0.05.

**Table 4:** Chemical constituent identification of rhizobial isolate ethyl acetate extract by GC-MS.

No	Retention Time	Compound	Area %	Molecular Formula	Molecular Weight
1	12.47	Tetradecane, 2,6,10-trimethyl-	0.57	C_17_H_36_	240
2	13.29	15-methyltricyclo [6.5.2(13,1 4).0(7,15)] pentadeca-1,3,5,7,9,1 1,13-heptene	4.13	C_16_H_14_	206
3	16.50	Heptadecane, 2,6,10,15-tetramethyl-	1.27	C_21_H_44_	296
4	20.35	7,9-Di-tert-butyl-1-oxaspiro(4,5)dec a-6,9-diene-2,8-dione	4.19	C_17_H_24_O_3_	276
5	24.39	Oleic Acid	20.23	C_18_H_34_O_2_	282
6	25.82	1-Hexadecanol, 2-methyl-	4.48	C_17_H_36_O	256
7	29.20	1,2-benzenedicarboxylic acid	1.77	C_24_H_38_O_4_	390
8	31.62	2,2,3,3,4,4 hexadeutero octadecanal	0.58	C_18_H_30_D_6_O	274
9	34.72	4H-1-Benzopyran-4 one, 2-(3,4-dimethoxyphenyl)- 3, 5-dihydroxy-7- methoxy-	5.66	C_18_H_16_O_7_	344

## Data Availability

Not applicable.

## Supplementary Materials

The following supporting information can be downloaded at: https://www.mdpi.com/article/10.3390/plants12010045/s1, Figure S1: Synthesis of silver nanoparticles by *Rhizobium leguminosarum* supernatant.
