# Supplementary material for: Rhizobium leguminosarum bv. viciae-Mediated Silver Nanoparticles for Controlling Bean Yellow Mosaic Virus (BYMV) Infection in Faba Bean Plants
Source: Plants (Basel). 2022 Dec 22;12(1):45. doi: 10.3390/plants12010045 (PMC9823325; doi:10.3390/plants12010045)
Supplement: Supplementary file 1 [file plants-12-00045-s001.zip › plants-2042583-supplementary.pdf]

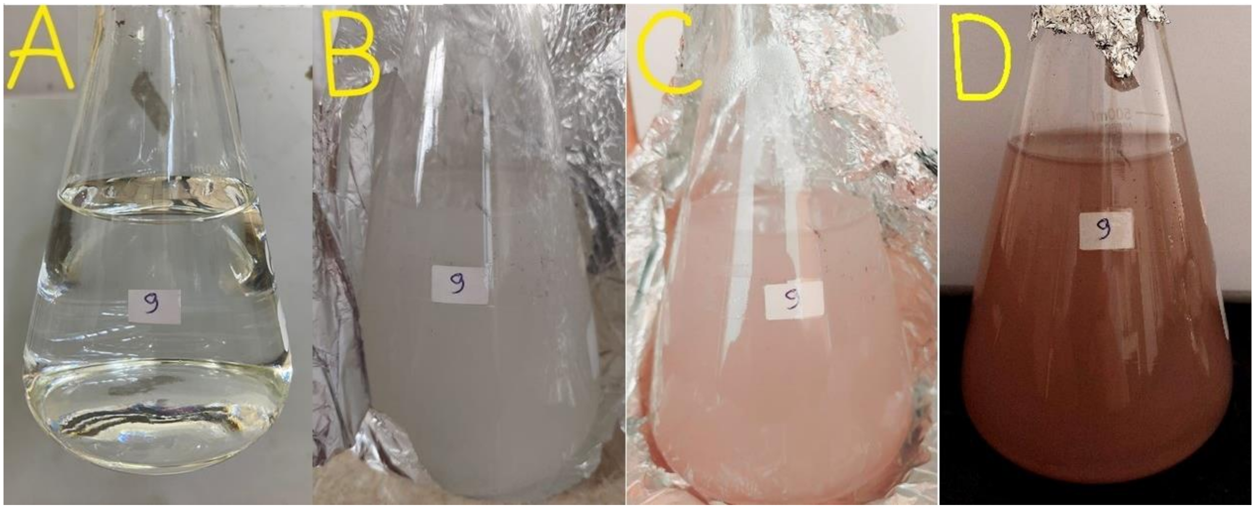

Figure S1. Synthesis of silver nanoparticles by *Rhizobium leguminosarum* supernatant A, silver nitrate in distilled water (control); B, silver nitrate in distilled water with *Rhizobium* media-free; C, silver nanoparticles after 24 h of incubation with supernatant (whitish rose); D, silver nanoparticles made after 96 h (yellowish brown)
